# A controlled pilot intervention on community violence prevention, financial and social capital generation in Dar Es Salaam, Tanzania

**DOI:** 10.1186/s12889-022-12723-x

**Published:** 2022-02-17

**Authors:** Louis Jansen, Anne H. Outwater, Michael Lowery Wilson, Masunga K. Iseselo, Till Bärnighausen

**Affiliations:** 1 Heidelberg Institute of Global Health, Ruprecht-Karls-University, Postal address: Im Neuenheimer Feld 130/3, 69120 Heidelberg, Germany; 2 Department of Community Health Nursing, Muhimbili University of Health and Allied Sciences, Dar es Salaam, Tanzania; 3 Injury Epidemiology and Prevention (IEP) Research Group, Turku Brain Injury Centre, Department of Clinical Neurosciences, Turku University Hospital, University of Turku, Turku, Finland; 4 Department of Clinical Nursing, Muhimbili University of Health and Allied Sciences, Dar es Salaam, Tanzania

**Keywords:** Community violence, Economic empowerment, Social capital, Violence prevention

## Abstract

**Background:**

Community violence has been found to be highly prevalent in Dar es Salaam, Tanzania. Increasing socioeconomic inequality has been outlined as one of the main causes of community violence. This controlled pilot trial aimed at evaluating the impact of beekeeping and entrepreneurship training on community violence exposure, financial and social capital generation, and employment structure.

**Methods:**

Poisson regression was used to compare pre- and post-intervention risk ratios for community violence exposure. Linear regression was used to depict change in weekly income and utu scores. Employment rate structures were determined pre- and post-intervention.

**Results:**

This study reports that compared to the Control arm beekeeping and entrepreneurship training appears to have protected young men in Dar es Salaam from exposure to community violence (All = 0.62 (0.40–0.96), Beekeeping = 0.57 (0.30–1.08), Entrepreneurship = 0.62 (0.33–1.17)), while increasing financial (All = 23,145 (− 27,155 – 73,444), Beekeeping = 29,310 (− 26,079 – 84,698), Entrepreneurship = 82,334 (12,274 – 152,293)) and partially also social capital (All = − 0.24 (− 1.35–0.87), Beekeeping = 0.85 (− 0.26–1.96), Entrepreneurship = 0.30 (− 1.16–1.77)). Financial dependency across all arms was reduced from 29.1 to 2.2%.

**Conclusions:**

Our study reports that beekeeping training and entrepreneurship seminars appear to have a protective effect against exposure to community violence among young men in Dar es Salaam, while partially also increasing financial and social capital, as well as reducing financial dependency. We recommend that these results should lay the foundation for an adequately powered randomized trial to confirm the study’s efficacy.

**Trial registration:**

retrospectively registered at ClinicalTrials.gov (Identifier: NCT04602416; October 26, 2020).

## Background

Community violence is a medico-social problem of public health importance worldwide. In the African region, only a few countries have systematically examined community violence and its etiology. Increasing reports of vigilantism and community violence in multiple African countries such as South Africa, Tanzania, Liberia, Ghana and Zimbabwe, have aroused significant concern over community well-being [1]. In South Africa for example, vigilantism is one of the most common forms of community violence, and one which is often directed towards the South African justice system [2]. A similar development has been reported in Liberia where vigilante groups form to protect their communities from those who are perceived to be social deviants [3].

In Dar es Salaam, Tanzania, community violence has been found to be highly prevalent. Tanzania experiences a high overall crime rate, with most of it being due to petty theft and burglary [4]. Community violence is a common reaction towards this kind of deviant behavior. In Dar es Salaam, the majority of homicides are carried out through community lynching and vigilantism [5]. A widespread perception of law enforcement being corrupt and inefficient, leads vigilante mobs to take matters into their own hands. Increasing income inequality in combination with mistrust, have been outlined as two of the main causes of community violence [1, 6]. Previous research in Tanzania has revealed that unemployment or marginal employment is one of the most important variables in predicting violent death in Dar es Salaam [7].

As opposed to Tanzanian village settings, where differences in socio-economic inequality are often small, in the country’s largest city, Dar es Salaam, inequality is significantly more apparent [8]. Young men in particular experience the conflict between wider community expectations of providing economically for their families and themselves and their lack of relevant job skills and access to financial capital [9]. As education is costly, requires time and is perceived to not provide immediate financial benefit, young men in Dar es Salaam often resort to low-paid day labor or, out of desperation, to pickpocketing [4]. It has been shown that males are overrepresented as victims of community violence in Dar es Salaam representing around 93% of all homicide victims [7, 10]. Without access to opportunity or requisite skills, one way of reducing young men’s exposure may lie in creating environments which facilitate skills acquisition and entrepreneurship.

Besides financial capital, social capital under the Bantu philosophical concept of ‘utu’ may play a role in preventing community violence as well [10]. ‘Utu’ encompasses values as “truthfulness, respect, good manners, generosity, obedience, humility, attentiveness, love for people, relatives, friends, providing for those you are with to help raise their children well” [11]. Especially in the Swahili culture ‘utu’ is a social and ethical construct that guides interpersonal relations. The core values of ‘utu’, which are of religious and philosophical descent, construct a humanist framework that highly values kindness and reciprocity towards the ones you are with. The utu concept intertwines financial and social capital and its generation. An utu-ubuntu business model incorporates the protective role of the community as a safety net into establishing a successful individual business agency. It is presumed that sharing risks and collaborating with peers to foster economic well-being of the individual fosters a cohesive society [12]. The idea to reduce violence by building sustainable livelihoods and social capital has predominantly been employed to reduce intimate-partner violence (IPV). Of particular interest for this study are the “Pigs for Peace” and “Rabbits for Resilience” programs. These economic empowerment initiatives use traditional forms of assets (livestock) to reduce negative outcomes and build on the resilience of adults and young adolescents by creating an enabling environment [13, 14].

Alluding to that, a promising source of engagement and income appears to be the vitalization of the primary sector. Tanzania is the world’s 10th largest honey producer [15]. The estimated production potential is currently unmet, mainly as a result of a lack of training [16]. The Ministry of Natural Resources and Tourism estimates that the rate of exploitation is around 7%. Exports from Tanzania are in demand in many countries and regions such as the European Union, Tanzania’s main export target [15]. A beekeeping entrepreneurship initiative has been launched by the Ministry to transform traditional beekeeping into small-holder commercial production to provide individuals with independent access to not only national but also international honey markets. Besides the financial benefits of beekeeping as micro-business, previous studies have reported multiple further benefits of beekeeping such as nutritional and medical benefits as well as improved communal structures [17–19]. Moreover, effective beekeeping training represents a potentially resilient strategy for safeguarding livelihoods via sustainable agriculture [20].

The present study aimed at evaluating the effect of beekeeping training and an entrepreneurship seminar series on male exposure to community violence, financial and social capital (‘utu’) generation and employment structure.

## Methods

### Site

The United Republic of Tanzania is a lower middle income country in East Africa with approximately 56 million inhabitants [21]. The mean age of its citizens is 17.9 years, with a population growth rate of 2.7%. The percentage of those in informal employment is estimated to lie around 85%. Informality is much more present in Dar es Salaam than in other cities or the rural areas of Tanzania [22]. The population growth rate of Dar es Salaam is 5.6% making it one of the fastest growing cities in the world [23]. The growth is partially fueled by an influx of young unemployed males from rural areas hoping to find work within the city. One result of an unsustainable ratio of unskilled job seekers to few employment opportunities has been a rise in crime. Tanzania experiences one of the highest overall rates of petty crime in Africa, with most of it being due to theft and burglary [4].

### Population sample

**Fig. 1:**
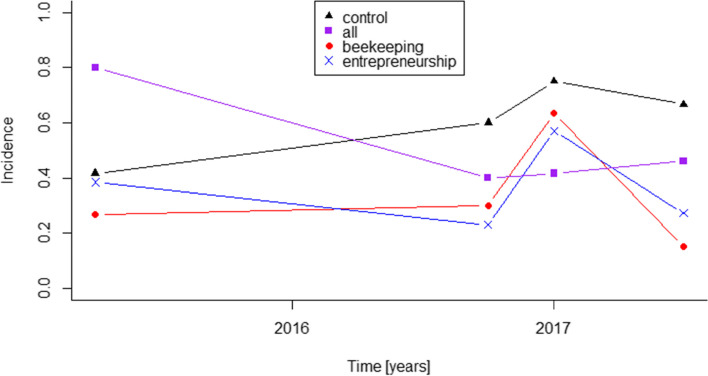
Incidence of community violence exposure of the four arms over the four study interviews

In Dar es Salaam, 55 men participated in the *RukaJuu!* study. They were selected from so-called urban ‘camps’, social gathering places that have formal leaders and members. These camps were described first in 2010 as appropriate venues for reaching at-risk youth. The camps have been verified and mapped within the four wards of Tandale, Manzese, Mabibo, and Mwanayamala [24]. For this study four camps out of 71 were selected (Fig. 1). The inclusion criteria for the camps were appropriate size (8–15 people), reassurance by the interviewers, that they felt safe enough to conduct the interviews, absence of weapons within the camp, number of male and female members, and whether or not the interviewer was told not to work there. Twenty camps were excluded because members reportedly carried weapons, five camps reported an unsafe environment, in nine camps the interviewer did not feel safe, and 23 camps had already been chosen for previous research. Of the remaining 14 camps eight had too many members and two leaders were not interested in participating. The remaining four camps were selected to take part in the *RukaJuu!* Study [25]. Each camp was randomly assigned to one study arm. We were informed that tragically one participant was killed for alleged petty theft during the study. Further information and graphical representation of the selection process can be found in Iseselo et al. (2019).

### Study design

The *RukaJuu!* pilot study consisted of four arms that were given different seminar and/or training schemes. All arms received an introduction and a health seminar. The Control arm received no further training, the All arm received entrepreneurship seminars and beekeeping training, the Beekeeping arm received training only on beekeeping, while the Entrepreneurship arm received solely entrepreneurship seminars. The data for this study were gathered via interviews conducted at four time periods. The baseline interview and the first session (Introduction) were held in April 2015. Further sessions and field sessions were held between June 2015 and July 2016. The three subsequent interviews were conducted in October 2016, January 2017, and July 2017. Before randomizing the camps into the four arms all participants were introduced to the project and were interviewed the first time (baseline interview). One participant turned 16 shortly after the introductory session (Birthday [DD/MM/YYYY]: 06/07/1999; Introduction: 24/04/2015). He only gave his own informed consent. All other participants were 16 or older at that time and also gave their own informed consent. The same questionnaire was used throughout all four interviews with the same questions being asked every time. A precise overview of the seminar and training schemes can be found in Iseselo et al. (2019). The sample then became a closed cohort that received two, six or ten day training sessions according to the arm their camp was randomized into. After the introductory session violence prevention was never presented as a topic.

The health sessions included advice on topics such as nutrition, communicable diseases, and first aid and was derived from United States Peace Corps medical training. The entrepreneurship training was based on the *Ruka Juu na Fema* TV show. The participants were shown videos that were used in a edutainment series aired on national television in 2011 and 2013 on the topics Sources of Capital, Saving and Investing for Profit, Business Plan, and Marketing/Customer Care [26]. In these sessions, coaches were attending training the sample in person on the topics. Lastly, the beekeeping training was led by beekeeping officers of the Tanzanian Ministry of Natural Resources and Tourism on the topics Beginning Beekeeping, Building Beehives, Hive Placement, and Harvesting. The latter three sessions were conducted in the field in a government forest near Kongowe, around 40 km west of Dar es Salaam.

The participants did not receive any financial compensation other than two meals per day-long session and busfare and material to construct beehives [25]. No start-up capital was provided for two reasons. Firstly, the sample was unexperienced in running a business so financial support might have been unsustainable over time. Secondly, we did not want to divide the different study arms as some group members knew each other and financial support for one arm could have led to resentment in the others.

### Measurements

Community violence exposure was assessed through the question: “Have you been exposed to violence in the community?”. The dependent variable community violence exposure was based on the World Health Organization (WHO) typology of interpersonal violence [27]. It specifically included exposure to all forms of community violence as physical, sexual, psychological, and deprivation or neglect as all forms of violence ranging from minor to severe acts harm victims in individual ways. Moreover, the presence of interpersonal violence promotes a violent environment and normalizes its use [28]. All incidents [29] of family/partner violence were excluded from this analysis. The participants were also asked to report the month and year of these incidents. Only incidents within one year prior to the study up until the last interview were included in our analysis. All cases of exposure to community violence, as victim, perpetrator, or witness, were included since previous studies showed that perpetrators and victims share similar sociodemographic qualities and usually circulate the same milieus sometimes having experienced violence both as perpetrator and victim [30]. Double entries were only counted once. Application of these criteria resulted in 81 cases of exposure to community violence. Participants were asked to state their last week income prior to each interview, which resulted in the variable ‘weekly income. Participants were also asked to state their current employment. Their answers were coded into six categories as seen in Table 1.

**Table 1:** Coding of current employment status

Answers	Category
Unskilled work, day labour	Not trained
Unemployed, searching for work, dependent	Dependent
Driver (motorcycles, cars, bus conductors)	Driver
Microbusiness	Microbusiness
Semi-skilled, vocational training, boxing, having gone through at least a minimum of specialized training, hotelier, selling books	Minimally trained

To compute utu scores, we used the following four variables: dependents, religious attendance, helping others, and group activities. We derived the idea of an utu score from Kibusi et al. who had already operationalised the concept of utu [10]. The cut-off for religious attendance was chosen based on the results of Kibusi et al. They showed that religious attendance more than once per week was associated with a significantly lower risk for being violently killed in Dar es Salaam [10]. Having people depend on you is a fundamental part of the utu collective identity. This net is formed through relationships of rights and obligations that are based on sharing wealth and resources with one another. It provides the community with strength, security, and an identity. As a social net may buffer risks that lead to socioeconomic isolation we included helping others and participating in group activities in our score [31]. Table 2 shows the coding of the questions and how the score was calculated.

**Table 2:** Coding of items and calculation of utu scores

Variable	0 points (N)	1 point (N)
Religious attendance	Weekly, monthly, 2–3 times a year, never (173)	Daily (14)
Number of dependents	0 (58)	1–7 (129)
Helping others	No (45)	Any kind of help like food, money, daily needs, clothes, education, transport, health, funerals, charity, giving lifts (142)
Group activities	No, nothing specific (20)	Any kind of group activity like work related, social support, microfinancing groups (167)
Group activities (member of more than one group)	No, nothing specific (153)	Any kind of group activity like work related, social support, microfinancing groups (34)
Sum	0	5

### Statistical analysis

The aim of the analysis was to quantify the impact the three interventions had on community violence exposure, weekly income, utu score, and employment structure. Initially, we plotted three graphs depicting the development of community violence exposure rate, mean weekly income, and mean utu score within each arm over the four interviews. We used two modified Poisson regression models in a generalized estimating equations design (GEE) to calculate and compare pre- and post-intervention risk ratios for community violence exposure. To analyse weekly income and utu scores we calculated the individual change from the baseline interview to the last interview by subtracting the latter of the former (Change = Income/Score at T3 – Income/Score at T0). Linear regression models were used to depict the relationship between change in income or utu and the intervention arms. Since the participants of the four arms differed significantly in age, analyses were adjusted for mean-centred age. We also compared pre-intervention to post-intervention employment rates to analyse if the structure was altered by the different intervention schemes. All analyses were conducted using the R Statistical Environment for Windows 10 [32].

## Results

The four interviews of the 55 study participants resulted in 187 observations of which 44.4% affirmed having been exposed to community violence in the last year. The mean income over all observations was 40,807 Tanzanian Shilling (TzS), with eight missing observations. The mean utu score over all observations was 2.6 with no missing observations. As stated above one young man who participated in our study was tragically killed after the introductory session. Seven participants were travelling (ten missed interviews), four had to work (six missed interviews), two moved out of Dar es Salaam (six missed interviews), and eight participants did not complete an interview for unknown reasons (eight missed interviews). This resulted in an average of 3.4 interviews per participant. The Control arm consisted of twelve members, the All and the Beekeeping arm of 15, and the Entrepreneurship arm of 13. Table 3 depicts the participation in each arm over the course of the study. Five participants of the Control arm, four participants from the All arm, six participants from the Beekeeping arm and seven participants from the Entrepreneurship arm did not complete all four interviews.

**Table 3:** Participation of the four arms over the four study interviews

Arm	Baseline	Interview 1	Interview 2	Interview 3
Control	12	10	8	9
All	15	15	12	13
Beekeeping	15	10	11	13
Entrepreneurship	13	13	7	11

The mean age was 21.6 years at baseline ranging from 15 to 39 years and differed significantly between the four arms (*P* value < 0.001). At baseline, the participants of the Entrepreneurship arm were the oldest with a mean age of 26.9. The Beekeeping arm was the second oldest with a mean age of 21.4. The two youngest arms were the All and Control arm with mean ages of 18.9 and 19.3, respectively.

### Development of community violence exposure

Fig. 1 depicts the incidence of community violence exposure in the four arms over all four interviews. The incidence in the Control arm increased by 59.9%, while the incidence in the three intervention arms decreased. The incidence in the All intervention arm and also the Beekeeping arm decreased by 42.3% each, while the incidence in the Entrepreneurship arm decreased by 29.2%.

The results of the age-controlled pre- and post-intervention regression models are depicted in Table 4 as relative risk ratios (RRR) with corresponding 95% confidence intervals (CI).

**Table 4:** Results comparing age-controlled pre- and post-intervention relative risk ratios for community violence exposure within the 4 study arms

	Pre-intervention results		Post-intervention results	
Arm	RRR (CI)	P Value	RRR (CI)	P Value
Control	*Reference*	*Reference*	*Reference*	*Reference*
All	1.91 (0.93–3.91)	0.079	0.62 (0.40–0.96)	0.033*
Beekeeping	0.66 (0.23–1.95)	0.455	0.57 (0.30–1.08)	0.084
Entrepreneurship	1.05 (0.33–3.32)	0.936	0.62 (0.33–1.17)	0.139

### Development of financial capital

**Fig. 2:**
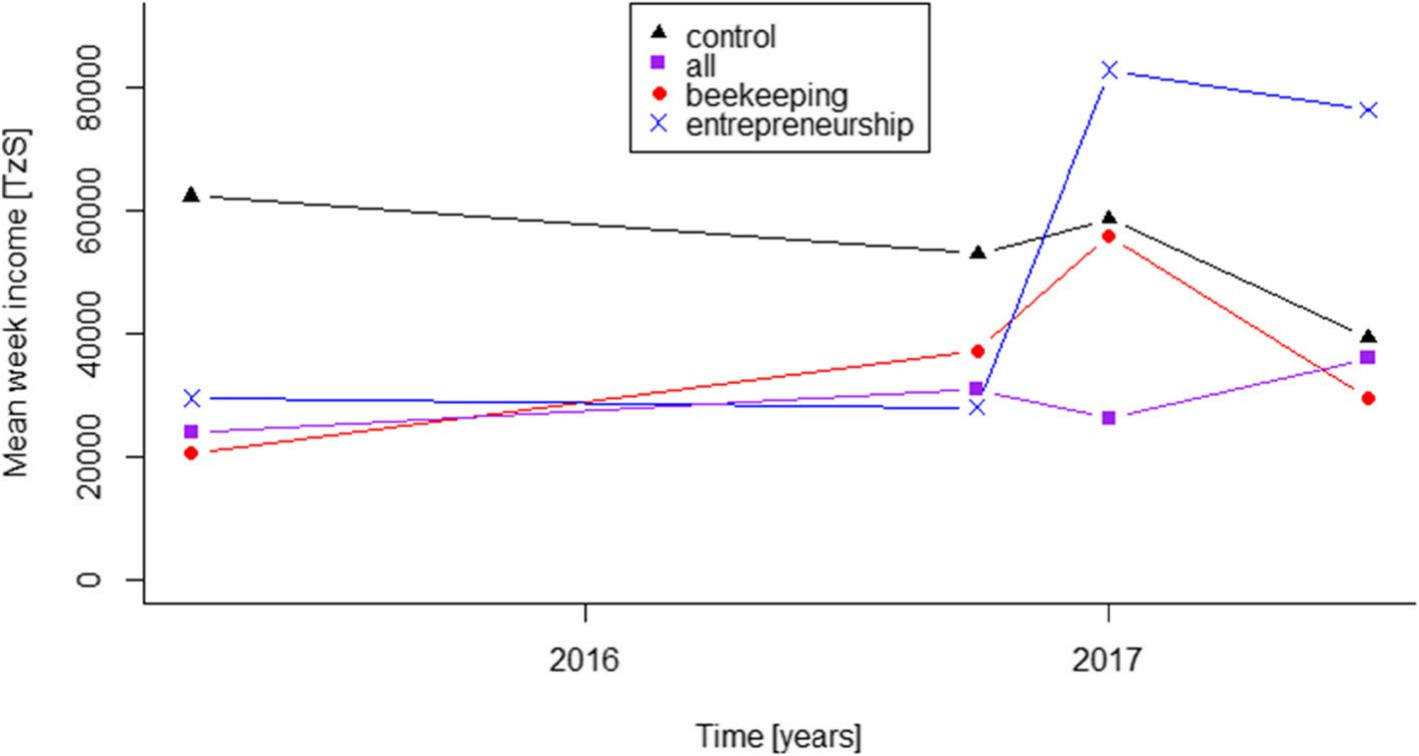
Mean weekly income of the four arms over the four study interviews

The development of mean weekly income in the four arms over time is shown in Fig. 2. The mean income of the Control arm decreased by 36.9% while the mean income of the All arm increased by 49.7% over the course of the study. Also, the mean income of the Beekeeping and Entrepreneurship arms increased by 43.7 and 158.3% respectively.

Two linear regression models were employed to compute confidence intervals and to control the results for baseline age. The control group was used as reference group. Table 5 shows a summarized analysis of these models.

**Table 5:** Results from the RukaJuu! Pilot study comparing crude and age-controlled change in week income (T3-T0)

Arm	Estimate (CI)	P Value	Controlled estimate (CI)	P Value
Control (reference)	−13,778 (− 51,943 – 24,388)	0.469	− 21,363 (− 63,599 – 20,874)	0.312
All	25,970 (−23,679 – 75,619)	0.296	23,145 (−27,155 – 73,444)	0.357
Beekeeping	24,444 (−29,530 – 78,419)	0.365	29,310 (−26,079 – 84,698)	0.290
Entrepreneurship	62,728 (10,120 – 115,335)	0.021*	82,334 (12,374 – 152,293)	0.022*

### Development of social capital

**Fig. 3:**
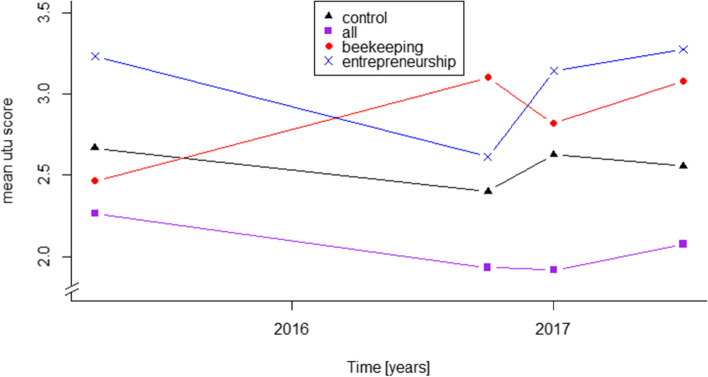
Mean utu scores of the four arms over the four study interviews

In Fig. 3 the developments of the mean utu scores in the four arms are displayed. The score of the Control arm decreased by 4% while the All arm decreased its score by 8%. The Beekeeping arm increased its score by 25%, and the Entrepreneurship arm by 1%.

As for financial capital, we also employed two linear regression models for utu score change to compute confidence intervals and control the results for baseline age. The control group was used as reference group. Table 6 shows a summarized analysis of these models.

**Table 6:** Results from the RukaJuu! Pilot study comparing crude and age-controlled change in utu scores (T3-T0)

Arm	Estimate (CI)	P Value	Controlled estimate (CI)	P Value
Control (reference)	−0.11 (− 0.95–0.73)	0.791	−0.22 (− 1.15–0.70)	0.626
All	−0.20 (− 1.29–0.90)	0.718	−0.24 (− 1.35–0.87)	0.666
Beekeeping	0.80 (− 0.29–1.90)	0.145	0.85 (− 0.26–1.96)	0.129
Entrepreneurship	0.02 (− 1.11–1.17)	0.971	0.30 (− 1.16–1.77)	0.542

### Development of employment structure

In Table 7 the pre- and post-intervention employment rate structures within the different sectors are displayed. In all three intervention arms the rate for being financially dependent dropped to zero, while it also declined in absolute and relative figures in the Control arm.

**Table 7:** Pre- and post-intervention (T0 & T3) employment rate structures within the different sectors

Pre-intervention [N (%)]					
Work	Control	All	Beekeeping	Entrepreneurship	Total
Not trained	2 (16.6)	4 (26.7)	6 (40.0)	4 (30.8)	16 (29.1)
Dependent	3 (25.0)	7 (46.7)	3 (20.0)	3 (23.1)	16 (29.1)
Driver	4 (33.3)	3 (20.0)	2 (13.3)	0 (0.0)	9 (16.4)
Microbusiness	1 (8.3)	1 (6.7)	3 (20.0)	1 (7.7)	6 (10.9)
Minimally trained	2 (16.6)	0 (0.0)	1 (6.7)	5 (38.5)	8 (14.5)
**Post-intervention [N (%)]**					
Not trained	2 (22.2)	5 (38.5)	7 (53.8)	3 (27.3)	17 (37.0)
Dependent	1 (11.1)	0 (0.0)	0 (0.0)	0 (0.0)	1 (2.2)
Driver	4 (44.4)	3 (23.1)	2 (15.4)	0 (0.0)	9 (19.6)
Microbusiness	1 (11.1)	2 (15.4)	4 (30.8)	4 (36.4)	11 (23.9)
Minimally trained	1 (11.1)	3 (23.1)	0 (0.0)	4 (36.4)	8 (17.4)

## Discussion

This study evaluates the effect of a beekeeping and entrepreneurship training series on male exposure to community violence, financial and social capital (‘utu’) generation and employment structure in Tanzania. The associations reported indicate that the three interventions predict a lowered community violence exposure risk among males in Dar es Salaam compared to the Control arm. It was also shown that the mean week income of all three and mean utu scores of two intervention arms, Beekeeping and Entrepreneurship, increased. Lastly, the percentage of financially dependent participants decreased from 29.1% at baseline to 2.2% after 12 months with only one participant from the Control arm being financially dependent at last. The findings contribute to and extend a small sample of empirical literature on the efficacy of economic beekeeping programs [16, 20, 33]. A qualitative analysis of this study already found that the interventions had led to increased customer care and social capital, as well as improved financial management [25]. There is evidence that access to social capital such as utu can protect from and prevent violence, while a loss of access paired with inequalities increases rates of violence among young adults [34–36]. Employing beekeeping to restore access to social capital in communities that were deprived of it is a relatively new idea. In Rye Hill Prison in Warwickshire, England, inmates were taught the principles of beekeeping. An assessment of the initiative by Coventry University concluded that the participants had a common bond and felt part of something greater than their own role. Hence, a community spirit was created [19]. The link between horticulture and health and wellbeing has been established through several studies showing that gardening and food growing has a positive impact on physical activity, mental relaxation and stimulation [18, 33]. Moreover, it has been concluded that nature based and social engagement positively affects health and encourages the acceptance of a healthier way of life [37]. These beneficial health effects of agriculture as well as the favorable social component of beekeeping may have caused the increased utu scores as well as a lower risk for community violence exposure in the Beekeeping arm. Comparable effects, albeit on IPV, have been found in the “Pigs for Peace” and “Rabbits for Resilience” studies. The improvement of social and financial capital through a productive asset transfer program led to reduced IPV victimization and perpetration [13]. Combining an economic empowerment initiative with responsibility for living animals as pigs, rabbits, or, as in this study, bees, ties economic success to caregiving. By sharing skills such as beekeeping and working not only to improve one’s individual situation but also to improve the well-being of others, the transition from a violent towards a supportive micromilieu may be achieved [38]. The aforementioned principles of an utu-ubuntu business model that promotes caregiving structures to foster economic success are found in these actions [12]. Economic empowerment to reduce violence exposure, especially IPV, has been the focus of various studies [39, 40]. Entrepreneurship training and start-up capital are a usual feature in those studies and there have been mixed effects on IPV rates. Simultaneously, those two factors are frequently viewed upon as the most common hurdles when trying to run a business [41]. However, our intervention only provided entrepreneurship training and no start-up capital. There has been some evidence that entrepreneurship training can be more effective than subsidies in running a successful business [42]. It appears as if the development of an entrepreneurial culture promoting business creation and development has benefited the participants of our Entrepreneurship arm, as shown by the significant change in week income. Also, the Entrepreneurship arm recorded the biggest relative and absolute rise in the employment category “microbusiness”. It was outlined that petty theft and pickpocketing are predictors of community violence in Dar es Salaam. These acts usually happen out of motives like hunger and family responsibilities, while the perpetrators are well-aware of the possibly life-threatening consequences of their actions [4]. These acts are also perceived as antisocial and harmful to society [43]. By improving entrepreneurial skills, the participants lay the foundation for self-sufficient income generation in a non-violent micromilieu. Hence, they should not have to engage in high-risk behaviour such as petty theft anymore. The significantly lower risk for community violence exposure compared to the Control arm supports this theory.

The combination of the two interventions, beekeeping and entrepreneurship, resulted in a significant protective effect on community violence exposure. The effect size was similar to those observed in the two other intervention arms. Like in the Beekeeping arm we detected a non-significant rise in week income. Also, comparing pre- and post-intervention employment rates, all seven of the 15 participants of this arm, who were financially dependent at the start of the study, gained some form of employment. However, contrary to the other two intervention arms the utu score of the All intervention arm fell, over the course of the study. At baseline the All intervention arm was the youngest arm and had the highest rate of community violence exposure. In addition to that their incomes were the second lowest and their utu scores were the lowest of the four arms. We hypothesize that the combination of these more adverse circumstances may have prevented possible improvements of their utu scores. Ultimately, we cannot causally explain why their scores did not increase in the way the two single interventions arms experienced.

This study provides valuable insight into the prevention of community violence in combination with financial and social capital generation in an urban African setting as Dar es Salaam. The results suggest that short training interventions to increase beekeeping and entrepreneurial skills may decrease exposure to community violence. Furthermore, we could report a significant increase of weekly income through entrepreneurship seminars as well as a non-significant increase of utu, a concept of social capital, in the Beekeeping arm. Despite that, our findings also have the following limitations since it also remains confined on several topics.

This pilot trial only examined a small non-randomized sample. In the past randomization of at-risk populations in Dar es Salaam proved difficult in practice due to safety concerns and high drop-out rates. The selection process of the camps may have altered the findings as a certain type of camp was selected [24]. This study was conducted as a pilot trial, that was not adequately powered to detect a certain effect as no a priori power calculations were executed. These a priori power assessments were also not desired but have to be considered and calculated when implementing the findings in a larger randomized controlled trial [44]. Also, the answers to the questions asked were self-reported, which may have been subject to social desirability and non-response bias.

Today and in the future, low- and middle-income countries in socioeconomic transition like Tanzania are facing numerous hurdles that, if not overcome, may affect the well-being of the populations at-risk as examined in this study. In the light of the United Nations Sustainable Development Goals a wholistic approach to violence prevention within its own micromilieu is necessary to achieve future success. After abandoning the Ujamaa economic policies of Julius Nyerere, that were based on the concept of ‘utu’, but also involved widespread state intervention, which had the effect of depressing wages and increasing inflation, Tanzania faced a difficult transition in the process of liberalizing and stabilizing its economy during the latter half of the 1980s and the 1990s. Today, roughly two thirds of the population still work in the primary sector, which only accounts for approximately 23.4% of the gross domestic product [21, 45]. In the long run the private sector appears to be vital to the growth of the Tanzanian economy, yet as of now neither the country’s infrastructure nor the educational and health system are fully prepared to shoulder the load [46]. Hence, preventing violence through economic empowerment of vulnerable groups will likely include an efficient combination of production and marketing as examined in this pilot study. We plan on pursuing all three intervention schemes in future research. Before upscaling and testing the trial on a larger population we recommend analyzing the combined qualitative and quantitative insight on this trial. Possible adjustments include a discussion on start-up capital, defining process indicators for beekeeping, and further investigation of how the investigated variables interact. Other potential ways forward may be an adaption of these results for future violence prevention as IPV prevention programs.

## Conclusions

In conclusion, our study reports that beekeeping training and entrepreneurship seminars appear to have protected young men in Dar es Salaam from exposure to community violence through financial and social capital generation. Entrepreneurship seminars showed a significant change in weekly income, while beekeeping training increased the participants social capital scores, so called utu scores, the most. Our discussed findings also propose a future in-depth examination of the possible protective role of financial and social capital on community violence exposure. We recommend that our results should lay the foundation for an adequately powered and refined randomized trial to confirm the efficacy of the interventions.

## Data Availability

The data that support the findings of this study are available from the corresponding author upon reasonable request.
